# The tumor stroma influences immune cell distribution and recruitment in a PDAC-on-a-chip model

**DOI:** 10.3389/fimmu.2023.1155085

**Published:** 2023-05-02

**Authors:** Marlene Geyer, Lisa-Marie Gaul, Sabrina Luigia D`Agosto, Vincenzo Corbo, Karla Queiroz

**Affiliations:** ^1^ Mimetas B.V., Oegstgeest, Netherlands; ^2^ Department of Diagnostic and Public Health, University of Verona, Verona, Italy

**Keywords:** PDAC, microfluidics, immune cell infiltration, organ-on-a-chip, immuno-oncology

## Abstract

The dense tumor stroma of pancreatic ductal adenocarcinoma (PDAC) and its secreted immune active molecules provide a barrier for chemotherapy treatment as well as for immune cell infiltration to the tumor core, providing a challenge for immunotherapeutic strategies. Consequently, the investigation of processes underlying the interaction between the tumor stroma, particularly activated pancreatic stellate cells (PSCs), and immune cells may offer new therapeutic approaches for PDAC treatment. In this study, we established a 3D PDAC model cultured under flow, consisting of an endothelial tube, PSCs and PDAC organoids. This was applied to study the role of the tumor microenvironment (TME) on immune cell recruitment and its effect on partly preventing their interaction with pancreatic cancer cells. We observed that stromal cells form a physical barrier, partly shielding the cancer cells from migrating immune cells, as well as a biochemical microenvironment, that seems to attract and influence immune cell distribution. In addition, stromal targeting by Halofuginone led to an increase in immune cell infiltration. We propose that the here developed model setups will support the understanding of the cellular interplay influencing the recruitment and distribution of immune cells, and contribute to the identification of key players in the PDAC immunosuppressive TME as well as support the discovery of new strategies to treat this immune unresponsive tumor.

## Introduction

Immunotherapy has increasingly become a treatment option for various cancer types. However, PDAC is characterized by an immunosuppressive tumor microenvironment resulting in a challenging tumor to treat with currently approved immunotherapeutic strategies (1). Unsuccessful application of immunotherapies is likely related to the stroma secreted factors, hypoxia, desmoplasia and abnormal vasculature that favor the formation of an immunosuppressive infiltrate and prevent their interaction with cancer cells (2). Pancreatic stellate cells (PSCs) are predominant in the tumor stroma and exert a relevant role in secreting chemokines, cytokines, growth factors as well as extracellular matrix (ECM) components contributing to a denser tumor tissue (3). Activated PSCs, characterized by α-SMA expression, increase immune dysfunction, these also promote EMT and cancer cell invasion (4). Stroma-derived immunosuppressive molecules include IL-10, IL-6, IL-11, CXCL12, vascular endothelial growth factor (VEGF), transforming growth factor beta (TGF-β) and matrix metalloproteinases (5).

For immune cells to reach the tumor tissue, these first need to exit a blood vessel near the tumor and infiltrate the stroma. Upon receiving signals, these reach the tumor cells, which express tumor antigens to perform their antitumor responses. Stromal cells interact with immune cells through mechanical cues, shielding immune cells from reaching the tumor site and either physically trapping the immune cells upon direct cell-cell contact or chemically upon secretion of immune mediators (6). In addition, immune cells are mainly inactivated or rarely present, suggesting that the immune system is suppressed in PDAC (5, 7). Consequently, the cells are either in paucity and do not function well or are trapped in the tumor stroma unable to reach the tumor cells (8). Activated PSCs, therefore, seem to orchestrate several processes that together promote tumor growth as well as immunosuppression in PDAC.

Murine models have often supported developments in the field of tumor immunity and responses to its targeting. However, advances in cellular and microfluidic technologies are supporting the development of *in vitro* systems that potentially recapitulate key aspects of *in vivo* biology. These systems can be applied for dissecting the contribution of specific cell types as well as their interaction that support specific shaping of the immune microenvironment in diseased tissues (9, 10). In addition, immune migration studies have previously been done in transwells, where migration is gravity driven and likely not a response of immune cells to the formation of chemoattractant gradients (11, 12). Organ-on-chip systems are an alternative that allow 3D cultivation of multiple cell types, enabling cell-cell interactions, cell-matrix interactions, and flow (13, 14).

Considering that the PDAC stroma plays a key role in shaping the immune cell infiltrate, consequently limiting immune responses, we hereby developed and characterized a PDAC-on-a-Chip model to study the role of the endothelium and the stroma in immune cell migration. We envision that this model could provide a valuable understanding on immune cell infiltration in this tumor type as well as enable the development of new therapeutic approaches.

## Materials and methods

### Cell culture

PDAC organoids were acquired from a tumor resection performed with curative intent at the University and Hospital Trust of Verona. Written informed consent from the donors for research use of the tissue was obtained prior to acquisition of the specimens. Tissues for the generation of models were collected under protocol number 55859, approved by the local Ethics Committee (Comitato Etico Azienda Ospedaliera Universitaria Integrata) to V.C. (Prog. 3456CESC, 27/09/21). The organoids were cultured in a 6-well plate in 10 μl Matrigel (Corning^®^, 356231, 8.3-10.5 mg/ml) droplets. After seeding, the plate was placed in the incubator with bottom-side up for 15 min. For culturing, hCPLT medium was used, which consists of Advanced Dulbecco’s Modified Eagles Medium with Nutrient Mixture F-12 Hams – 500ml (Gibco, #12634-028), GlutaMax – 200mM (Gibco, # 35050-061), Hepes – 1M (Gibco, # 15630-080), Primocin – 50mg/ml (Invivogen # ant-pm-2), B-27 supplement (Gibco, # 17504-044), N-Acetylcysteine (NAC) (Sigma-Aldrich, # A9165-5G), Nicotinamide (Sigma-Aldrich, # N0636), hEGF (Gibco, # PMG8043), TGFβ Receptor inhibitor A83-01 (Tocris, # 2939), FGF10 (Peprotech, # 100-26), mNoggin (Peprotech, # 250-38), R-spondin-1 conditioned medium, Wnt3a conditioned medium, Gastrin (Tocris, # 3006) and Y-27632 Dihydrochloride (Sigma, # Y0503). The cells were harvested using Cell Recovery Solution (Corning^®^, 354253) and placed on ice for 30 min. The organoids were spun down at 300 g for 5 min, media was removed and incubated with TrypLE Express for 3 min (Gibco, # 12605-028) in the waterbath for enzymatic disruption. The organoids solution was spun down, resuspended in ice-cold media, counted and used.

PSCs (Klon 2.2) were obtained from Marburg University (15) and cultured in DMEM medium (Gibco, 10829-018) with 1% Penicillin/Streptomycin and 10% Fetal Bovine Serum (FBS). Cells were passaged at 80% confluency using Trypsin 2.5% (ThermoFisher, 15090046).

Human Umbilical Vein Endothelial Cells (HUVEC) (Lonza, C2519AS) were cultured in Endothelial Growth Medium (EGM-2) (Lonza, CC-3156) with 1% Penicillin/Streptomycin and 2% FBS (Gibco, 16140-071). The cells were seeded immediately after thawing.

PBMCs were derived from whole blood-derived buffy coats of healthy donors provided by Sanquin. Ficoll-Paque PLUS (15 ml) was added to a 50 ml Leucosep tube and centrifuged at room temperature (RT) for 30 min at 1000 x g. The blood-derived buffy coat was diluted 1:2 with sterile PBS and 25 ml of the diluted buffy coat was added to the Leucosep tube. The tube was centrifuged at RT for 30 min at 800 x g and plasma was carefully removed by aspiration. The PBMC layer was transferred to a 50 ml conical tube and cells were subsequently washed twice with PBS and centrifuged at RT for 10 min at 300 x g. Cell density was assessed with a cell counter.

After isolation, PBMCs were frozen and thawed upon use. For isolation of immune cell types, PBMCs were thawed and different immune cells isolated with their respective kits according to manufacturer’s protocol: EasySep Human B Cell Isolation Kit (StemCell Technologies, #17954), EasySep Direct Human T Cell Isolation Kit (StemCell Technologies, #19661), EasySep Human CD4+ T Cell Isolation Kit (StemCell Technologies, #17952), EasySep Human CD8+ T Cell Isolation Kit (StemCell Technologies, #17953), EasySep Direct Human Monocyte Isolation Kit (StemCell Technologies, #19669).

All cells were cultured in a humidified incubator at 5% CO_2_ and 37°C, and regularly tested for mycoplasma.

### OrganoPlate 3-lane

PDAC models were established in the OrganoPlate 3-lane (Mimetas, the Netherlands), a microfluidic platform, based on a 384-well plate format with 40 microfluidic chips. The OrganoPlate 3- lane consists of two perfusion lanes, and a middle lane used for ECM gel filling. Culture medium is added to the chips through the perfusion inlets and outlets. Cultures are monitored through the observation window.

In the OrganoPlate 3-lane, both Matrigel and Collagen type I (5 mg/ml, AMSbio, #3447-020-01) were used as ECMs. Collagen type I was neutralized in an 8:1:1 ratio with 1 M Hepes buffer (Gibco, #15630-056) and 37 g/L NaHCO3 (sigma, S5761) reaching a final concentration of 4mg/ml. The collagen mix was kept on ice and seeded within 10 min. PSCs were counted and the appropriate number of cells was resuspended in Collagen type I. ECM (2 μl) was loaded into the gel channel and incubated for 15 min at 37°C and 5% CO2 to allow for polymerization of the gel. After incubation, 50 μl of DMEM medium was added to the gel-inlet to prevent the gel from drying out. Next, HUVECs were seeded (10 000 cells per chip) in 2 μl of EGM-2 medium in the top perfusion channel, 50 μl of the same medium was added to the top medium inlet. The plate was incubated vertically in an angle of 75°, with the gel channels facing downwards to allow the cells to attach to the ECM gel for 2 hours. After 2 hours, 50 μl of medium was added to the outlets of the gel and top perfusion channel. Subsequently, PDAC organoids were resuspended in 2 μl Matrigel and seeded in the bottom perfusion channel. The plate was incubated for 15 min until hCPLT medium was added to the bottom lane onto the PDAC organoids compartment. After that, plates were placed in the incubator on a rocking platform (OrganoFlow, Mimetas, the Netherlands) at an inclination of 7° and an interval of 8 min.

### Barrier integrity assay

All media was removed from the plate and 80 μl/chip dye was prepared (FITC Dextran 150 kDa (Sigma, Cat#: 46946)). 20 μl of medium was pipetted into the gel and medium in- and outlets and 40 μl of the dye solution was added to the lane containing the endothelial tube in the inlet and 30 μl to the outlet. Leakage of the fluorescent dye from the lumen of the endothelial vessel into the rest of the chip was imaged using the ImageXpress XLS Micro (Molecular Devices). Images were taken for 14 minutes with a 2-minute interval. The images were analysed by extracting the average fluorescence values of the top perfusion channel divided by the average fluorescence value of the bottom perfusion channel for each chip and timepoint, determined in Fiji. The apparent permeability (P_app_) value (cm/s) was determined:


$$Papp=\frac{\Delta C\left(receiver\right)*V\left(receiver\right)}{\Delta t*A\left(barrier\right)*C\left(donor\right)}$$

ΔC_receiver_ is the difference between the fluorescence intensity measured in the bottom perfusion channel between t=0 and t=14 min, V_receiver_ is the volume of the measured region in the ECM channel, Δt is the time between start of the assay and endpoint (14 min), A_barrier_ is the surface of the ECM barrier with the upper perfusion channel (0.0057 cm^2^), and C_donor_ describes the fluorescence intensity measured in the top perfusion channel.

### TEER

The electrode board was prepared and the OrganoPlate was equilibrated at RT for 30min. The electrode board of the TEER device (Mimetas, the Netherlands) was placed on top of the plate and TEER measurement was performed.

### Transendothelial migration quantification

5 μM MitoTracker Deep Red was added to the PBMCs in AIM-V medium. The cells were incubated in the dark at 37°C for 30 min. After the incubation, PBMCS were washed with medium, resuspended in the appropriate volume of medium and seeded in the top lane of the OrganoPlate upon exchanging EGM-2 medium of HUVECs with a 50:50 mixture of AIM-V and EGM-2 containing the PBMCs.

Imaging of the migration was done with the ImageXpress® Micro XLS confocal microscope (Molecular Devices). Montages were created using Fiji. A migration quantification tool made in Fiji, specifically developed for the OrganoPlate 3-lane was used.

### Immunostaining

The content of the OrganoPlates was fixed with 3.7% Formaldehyde (Sigma, # 252549-1L) in HBSS (+Ca/Mg) (Sigma, # 55037C-1000ML) for 15 min. The plates were washed twice with PBS (Gibco, t# 70013065) for 5 min each. PBS was added to all chips, the plate was sealed and stored until used for immunostaining. For staining, the plates were kept on a rocking device during all incubation steps. The cells were first washed for 5 min with washing solution containing 4% FCS (Gibco/ATCC, cat# A13450) in PBS, permeabilized for 10 min with permeabilization buffer containing 0.3% Triton X-100 (Sigma, #T8787) in PBS and washed again for 5 min. The cells were then blocked with 2% FBS, 2% BSA (Sigma, # A2153) and 0.1% Tween20 (Sigma, # P9616) in PBS for 45 min. The primary antibody was prepared in blocking solution and added to the plate for 24h at RT. CD31 (Dako, #M0823) and ICAM-1 (Biotechne, #BBA3) were used in a 1:100 dilution. Alexa Fluor Plus 488 (Invitrogen, #A32723) was used as a secondary antibody in a 1:250 dilution. The secondary antibody was prepared in blocking solution and added to the plate after washing the plate twice for 3 min and incubated for 24 h. The plate was washed again twice for 3 min after incubation. The cells were washed with PBS once for 1 minute, incubated with NucBlue™ Live ReadyProbes™ Reagent (Hoechst 33342) (Invitrogen, R37605) and ActinGreen™ 488 ReadyProbes™ Reagent (Invitrogen, R37110) and the plate was filled with PBS, sealed and kept in the fridge until imaging.

### Live and dead assay

Calcein-AM (Lifetechnologies, #C3099), NucBlue Live ReadyProbes Reagent (Life technologies, #R37610) and DraQ7 (BioStatus, #DR71000) were used for staining live and dead cells and the nucleus. The reagents were added to the medium and distributed to the perfusion inlets and outlets. The mix was incubated for 45 min on the rocker and fluorescent imaging was performed.

### Luminex

Medium was sampled from the OrganoPlate and added to the pre-ordered plate containing the analyte-specific capture antibodies, which bind to the analytes of interest according to the kit protocol. The Human Magnetic Luminex^®^ Assay was used (bio-techne, #LXSAHM-11). Samples were analyzed on the MAGPIX xPONENT^®^ software. The samples were normalized and compared to standards.

### Stromal targeting

For targeting the stroma, several compounds were added as 2 µM solutions to the OrganoPlate after medium was removed on day 4. The following compounds were used: Halofuginone (MedChem Express, #HY-N1584) as a PSC/CAF and SMAD 2/3 inhibitor; Galunisertib (Selleck Chemicals, #S2230) as TGF-β receptor inhibitor; Vismodegib (MedChem Express, #HY-10440) as a SHH pathway inhibitor, Pirfenidone (MedChem Express, #HY-B0673) as a cell cycle inhibitor of CAFs; and Losartan (MedChem Express, #HY-17512) as a TGF-β ligand inhibitor (16).

### Statistical analysis

All statistical analyses were conducted in GraphPad Prism version 9 (GraphPad Software, San Diego, CA, USA) and data was presented as mean ± standard deviation (SD). Differences in survival were assessed using one-way or two-way ANOVA in combination with respective Tukey’s multiple comparison test or Sidak`s multiple comparison. Luminex data was analysed using Kruskal-Wallis test, correction for multiple comparisons by Dunn’s test. A statistical significance of p ≤ 0.05 was maintained. The significances are shown as asterisks in the figures (* = p< 0,05; ** = p< 0,01; *** = p< 0,001; **** = p< 0,0001). Independent experiments are denoted by N, while replicates per experiment are denoted by n. Sample size was chosen based on the variation and standard deviation between samples to ensure significance of the data. F-tests, descriptive statistics and row analysis were performed to ensure similar variance between the groups.

## Results

### Development of a PDAC tumor microenvironment on-a-Chip

**Figure 1:**
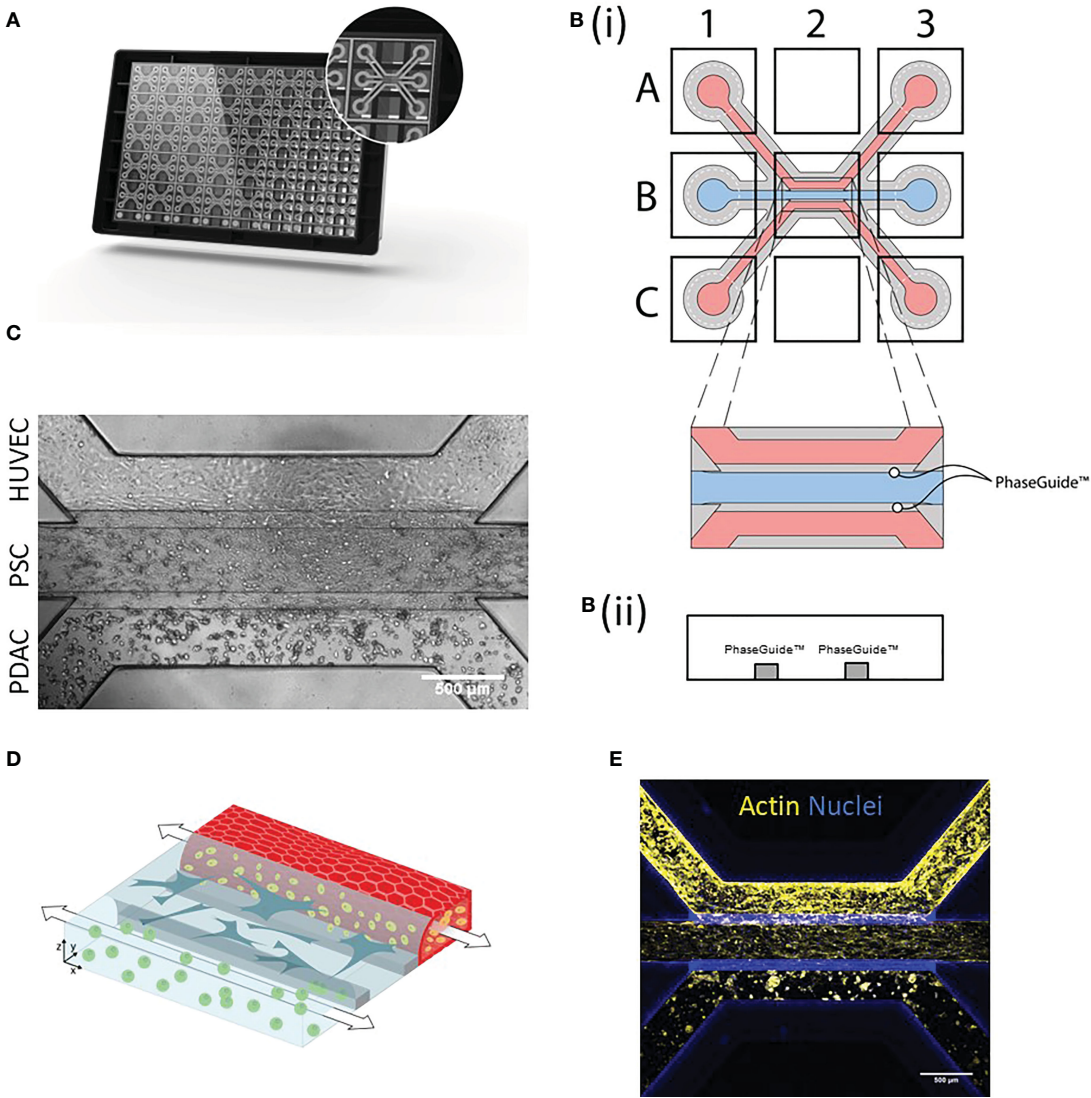
PDAC tumor microenvironment-on-a-Chip: **(A)** The OrganoPlate 3-lane comprises 40 chips with 3 microfluidic channels each. **(B)**(i). Endothelial cells were seeded into the top lane (A1) and due to capillary forces, the cells were distributed throughout the lane (A3) and allowed to form a tube. PSCs were loaded into the middle lane (B1) and PDAC organoids into the bottom lane (C1 distribution to C3). **(B)**(ii) Side view of a chip comprising the three lanes with PhaseGuides, that allowed for the compartmentalization of different cell types. Microscopic images were acquired from the observation window, which comprises all three lanes (B2) and which can be seen in **(C)** Phase contrast image shows culture organization in an OrganoPlate 3-lane chip. 4x acquisition, Scale bar=500 µm. Images acquired on the ImageXPress Micro XLS Widefield High-Content Analysis System® (Molecular Devices). **(D)** Schematic representation of the 3D culture, where PBMCs (yellow) migrated from the HUVEC tube (red) through the stroma (blue) to PDAC organoids (green). **(E)** Immunostaining with Actin (yellow) and NucBlue (blue). The cells were imaged on the confocal microscope. Shown are 4x maximum intensity projections, imaged on the ImageXpress Micro Confocal (Molecular Devices). Scale bar= 500 µm.

The stroma is considered the major player in shaping the PDAC immune microenvironment. In order, to recapitulate cellular interactions observed in PDAC tumors, we developed a microfluidic-based PDAC model and subsequently applied it in immune migration studies. PDAC organoids and PSCs were characterized and model setups were established in the OrganoPlate 3-lane (**Figures S1**, **1A**, **B**). These consist of PDAC organoids, PSCs, endothelial and immune cells. First, the middle lane was seeded with Collagen I type ECM containing PSCs. Subsequently, the top lane was seeded with endothelial HUVEC cells, which self-assembled into a tubule under flow conditions. PDAC organoids were cultured in Matrigel in the bottom lane (**Figure 1C**). All cell types were seeded on the same day. After cell seeding, cultures were then placed onto an OrganoFlow for perfusion, which enabled nutrient distribution and waste removal (**Figures 1D, E**). Cultures were allowed to develop for 4 days. For migration studies, the HUVEC tubule was loaded with CMRA labeled PBMCs and the migration towards the PDAC organoids was followed for 72h, cultures were imaged every 24h. Migration of PBMCs was analyzed using an in-house cell counting tool developed in FIJI.

This model setup and variations of it were further used to investigate the role of the tumor microenvironment, particularly the endothelium and PSCs, on immune cell recruitment in PDAC.

### Influence of the endothelial inflammatory status in PBMC migration

Inflammation occurs in response to tissue damage and cancer, which usually results in vascular activation and increased recruitment of immune cells towards the site of inflammation (17). Vascular responses such as changes in barrier function were studied in inflamed HUVEC (exposed to 2.25 ng/ml TNFα) as well as in presence of a PDAC tumor compartment.

**Figure 2:**
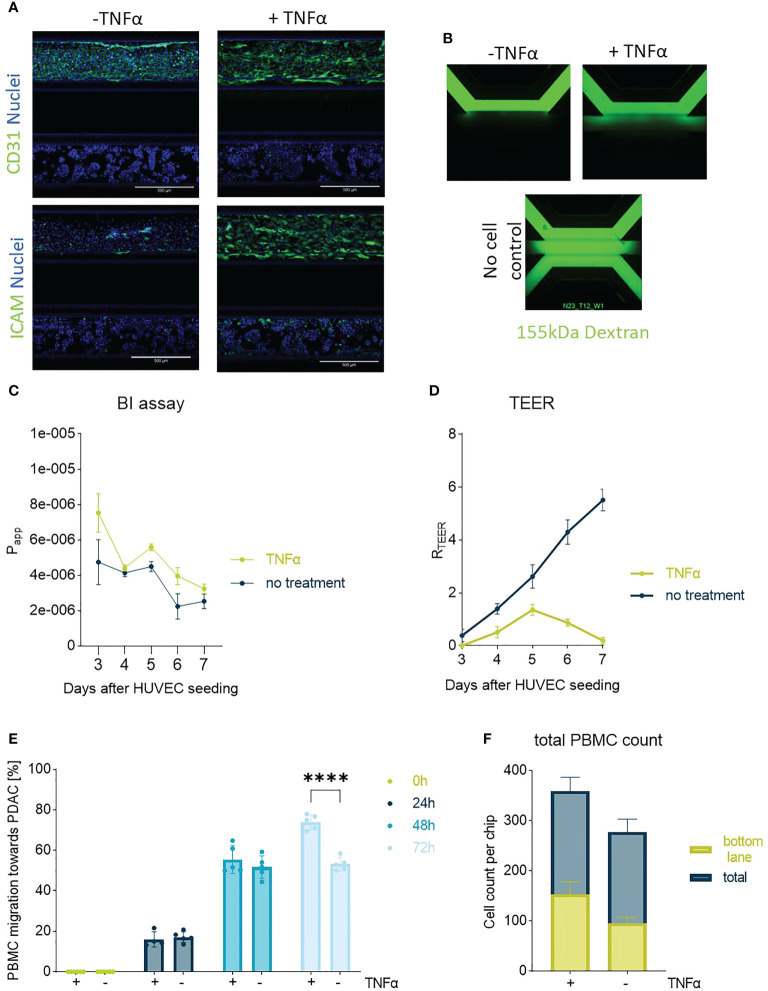
Endothelial barrier function and PBMC migration: In **(A)** one of the PDAC-on-Chip model setups is shown, and consists of a HUVEC tubule and PDAC organoids. Confocal imaging revealed the morphology of the HUVEC tube upon treatment with TNFα on day 3. The cells were stained on day 7 with CD31 (green), ICAM (green) and NucBlue (blue). Shown are 10x maximum intensity projections, imaged on the the ImageXpress Micro Confocal (Molecular Devices). Scale bar= 500 µm. **(B)** BI assay determined upon perfusion of a 155 kDa FITC-dextran. The dye diffused through the chip when no cells were present. 4x images acquired on the ImageXPress Micro XLS Widefield High-Content Analysis System® (Molecular Devices). **(C)** Barrier function was assessed with BI assays from day 4-7 after seeding upon perfusion of the chips with medium containing the 155 kDa FITC-dextran and corresponding apparent permeability (Papp) values were calculated. Shown are mean +- SD (N=3, n=3). **(D)** TEER measurements highlighted the role of TNFα in decreasing the barrier function of HUVEC tubes. (N=3, n=3) **(E)** Migration quantification of PBMCs towards PDAC organoids within 72h showed a slight influence of TNFα on PBMC migration behavior. Shown are mean+- SD (N=3, n=3) **(F)** Total cell count of PBMCs located in the chip compared to the number of cells that migrated to the bottom lane within 72h. Shown are mean+- SD (N=3, n=3).

HUVEC control tubules showed expected morphology as shown by CD31 immunostaining (18). In **Figure 2**, TNFα treated HUVEC tubules show clear increased ICAM expression (**Figure 2A**) and permeability (**Figures 2B–D**), consequently, leading to a better attachment of PBMCs and subsequent migration (**Figures 2E, F**).

**Figure 3:**
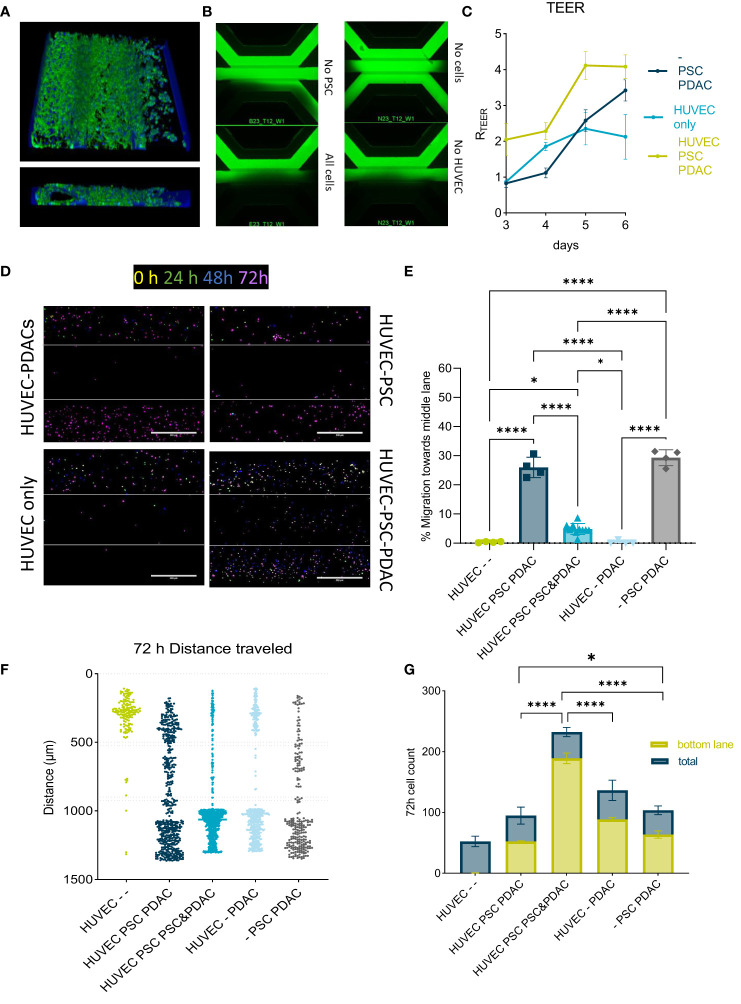
Migration of PBMCs in the PDAC TME model: **(A)** Top and side view of the 3D reconstruction of the complete model comprising a HUVEC tubule, PSCs and PDAC organoids. The cells were stained with Actin and NucBlue and imaged on the ImageXpress Micro Confocal (Molecular Devices) **(B)** BI-assay images of the tri-culture system. Perfusion of a 155k Da FIT-C labeled dextran, 4x magnification. Images acquired on the ImageXPress Micro XLS Widefield High-Content Analysis System® (Molecular Devices). **(C)** TEER measurements highlighted the role of PSCs in increasing the barrier function of HUVEC tubules (N=3, n=3). Statistical analysis revealed *p<0,05 for the - PSC PDAC sample and *** p<0,001 for the HUVEC PSCs PDAC sample compared to the HUVEC only control on day 6. **(D)** CMRA staining of migrating PBMCs imaged on the ImageXpress Micro Confocal (Molecular Devices) at 0h, 24h, 48h and 72h after PBMC seeding. 10x acquisition, Scale bar=500 µm. **(E)** Quantification of PBMC numbers in the middle lane. Migration was analyzed with a cell counting tool in FIJI based in confocal images. Shown are mean± SD (N=3, n=3). Data show % of cells that migrated compared to the total amount of cells counted in the chip. **(F)** Migration chart showing the position of single PBMCs and distance they traveled in the OrganoPlate after 72h in different setups. Migration was analyzed with a cell counting tool in FIJI based on confocal imaging. Shown are mean+- SD (N=3, n=3). **(G)** Total cell number of immune cells migrated towards PDACs within 72h versus total number of cells within a chip. Shown are mean+- SD (N=3, n=3).

However, HUVEC tubules presented a very poor morphology and a very high leakiness in response to TNFα, for this reason migration experiments were further conducted in absence of TNFα. Although, untreated HUVEC tubules in co-culture with PDAC organoids are more organized, these also present a poor barrier function in presence of PDAC organoids, therefore, recapitulating the leaky blood vessels observed in PDAC tumors (**Figures 2D**, **3B**).

### Effect of PDAC stromal barrier on immune cell distribution and migration

After confirming that the endothelium did not retain PBMCs or formed a good barrier in presence of PDAC organoids, the role of the PSCs was further evaluated. To bring more complexity into the model the HUVEC-PDAC model was expanded to include PSCs in the middle lane and create a HUVEC-PSC-PDAC model.

PSCs grew along the HUVEC tubule, building an additional barrier (**Figures 3A–C**). Cultures were characterized by actin and nuclei staining and respective 3D reconstruction images (**Figure 3A**) showed a stromal compartment formed alongside a fully developed HUVEC tubule. TEER and BI measurements in different model set ups indicated, that mainly PSCs were responsible for barrier formation (**Figures 3B, C**). Next, PBMCs were allowed to migrate in different culture setups. In presence of both PSCs and PDAC, 20-30% of PBMCs were retained in the stromal compartment (**Figures 3D, E**).

PBMC migration was not observed in HUVEC only controls (**Figure 3F**). PBMCs migrated within 24-72h in the different model setups tested. The highest numbers of PBMCs to reach the PDAC organoids compartment was observed in the model setup containing PDAC and PSCs in the bottom lane (**Figure 3G**).

### Soluble factors secretion changes in response to PDAC organoids and PSCs crosstalk

**Figure 4:**
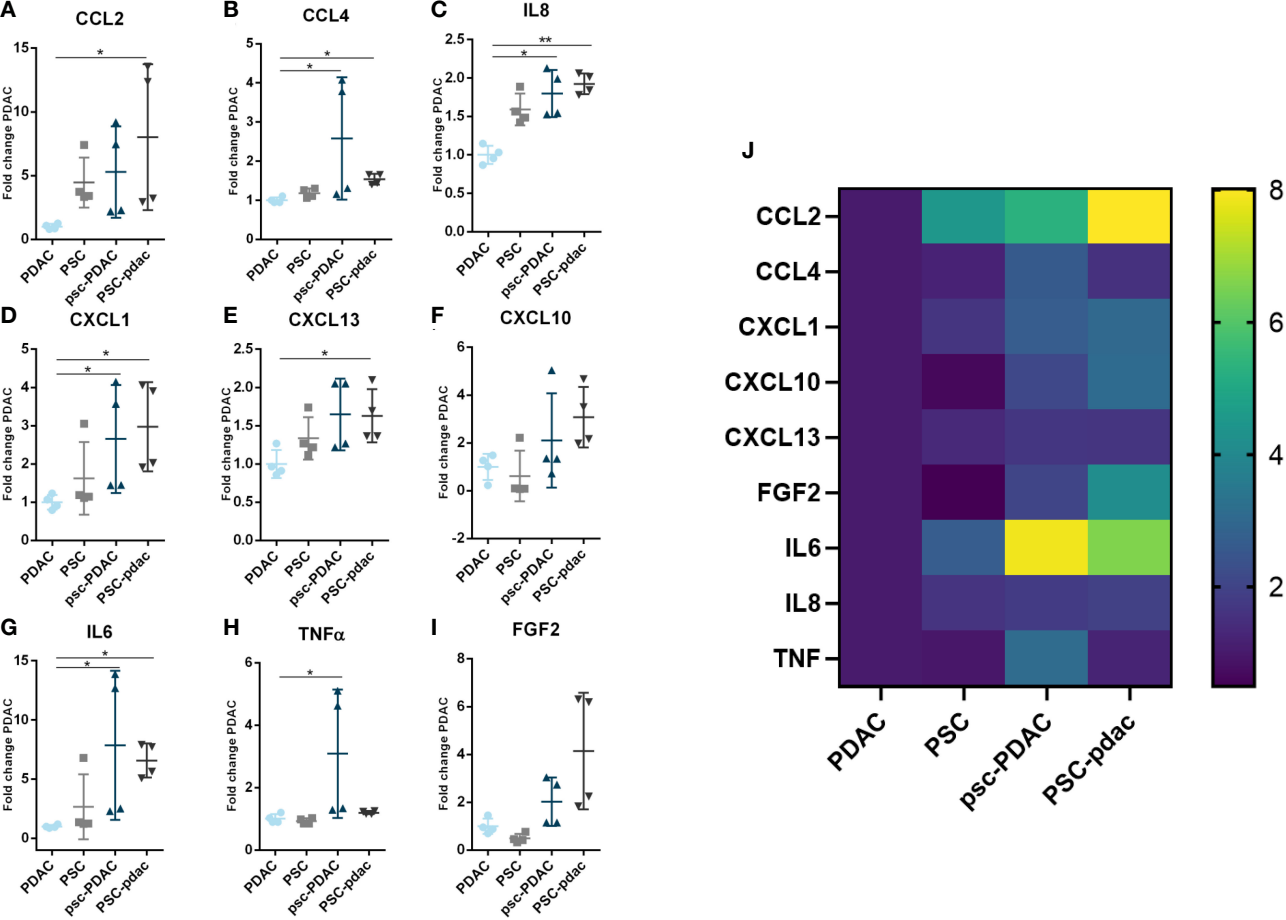
Soluble factors secretion: **(A–I)** Fold change of concentration of chemokines from the medium that was harvested from several culture setups. Samples were obtained from the following conditions: PDAC organoids grown in the bottom lane (in absence of PSCs), PSC grown in the bottom lane (in absence of PDAC, organoids), psc-PDAC samples were collected from the PDAC organoids grown in the bottom lane (in presence of a PSC compartment in the middle lane), PSC-pdac were collected from PSC grown in the middle lane (in presence of PDAC organoids growing in the bottom lane). **(J)** Heatmap of Luminex data for concentration fold changes. Luminex data was analyzed using Magpix. Samples were taken from the lane, that is depicted in capital letters. CCL2, CXCL1, CCL4, CXCL10, IL-6, IL-8, TNFα, CXCL13, FGF2, IFNy and IL-10 concentrations were measured in the supernatant (N=1,n=4).

To further explore the relevance of the stroma in attracting and influencing the distribution of immune cells in our model, chemokine, cytokine and FGF2 levels were determined in culture supernatants, collected from specific chip compartments, using a Luminex panel containing CCL2, CXCL1, CCL4, CXCL10, CXCL13, IL-6, Il-8, IL-10, TNFα, FGF2 and IFN-y (**Figure 4**).

Data are shown as fold change of PDAC organoids compartment in absence of PSCs. Almost all secreted factors were present in the supernatant of the different culture setups, except for INFy and IL10. These cytokines were present in very low concentrations and no significant differences were observed (data not shown). CCL2 and CXCL13 (**Figures 4A**, **E**) were increased in the PSC compartment in presence of PDAC organoids (adjacent channel) compared to PDAC organoids growing in absence of PSCs. TNFα was only increased in the PDAC organoids compartment of the co-culture (**Figure 4H**). CXCL1, CCL4, IL8 and IL6 were significantly increased in the PSC and PDAC compartments of the co-culture (**Figures 4B–D, G**). CXCL10 and FGF2 data showed a trend towards increased levels in the co-cultures (**Figures 4F, I**). FGF2 level was significantly increased in the PSC compartment of the co-culture in comparison to PSCs growing in absence of PDAC organoids (**Figure 4I**). Heatmap in **Figure 4J** summarizes the Luminex data and indicate that compartments of the co-culture setup show an increase in the secretion of most soluble factors measured. These results indicate that the biochemical microenvironment in our model is complex, and that the PDAC organoids-PSCs crosstalk lead to the increase of immune mediators and being those changes sometimes compartment dependent. In addition, these support the notion that PSCs in addition to a physical barrier, seem to shape a biochemical immune microenvironment as well.

### PBMC subtype preferential migration in a PDAC model

**Figure 5:**
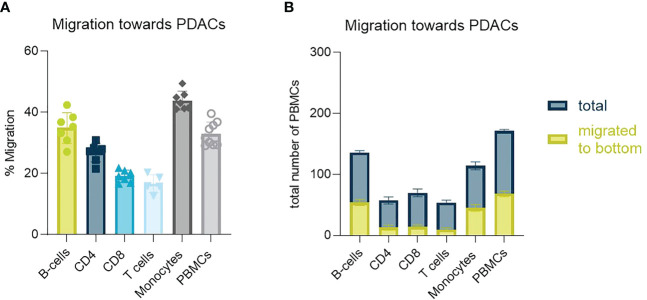
Identification of PBMC subtype migration **(A)** Migration percentage of total migrating T-cells, CD4+ T-cells, CD8+ T-cells, B-cells and monocytes towards PDAC organoids in a HUVEC-PDAC setup. Shown are mean+- SD (N=3, n=3). **(B)** Total cell number of diverse immune cell types migrated towards PDAC within 72h versus total number of cells within a chip. Shown are mean+- SD (N=3, n=3).

PSCs seem to function as a physical barrier as well as contribute with secreted factors that influence PBMCs migration and distribution *in vitro* preventing them in part from reaching the tumor cells. We next characterized the migratory behavior of isolated PBMC subtypes. T-cells were least likely to migrate towards PDAC organoids, whereas monocytes and B-cells were most likely to migrate to this compartment (**Figure 5A**). Total cell numbers showed, that around 40% of the total B-cell and monocyte population seeded, migrated towards to the organoids, whereas this percentage was much lower for T-cells (**Figure 5B**).

### Influence of stromal targeting on barrier function and immune cell infiltration

**Figure 6:**
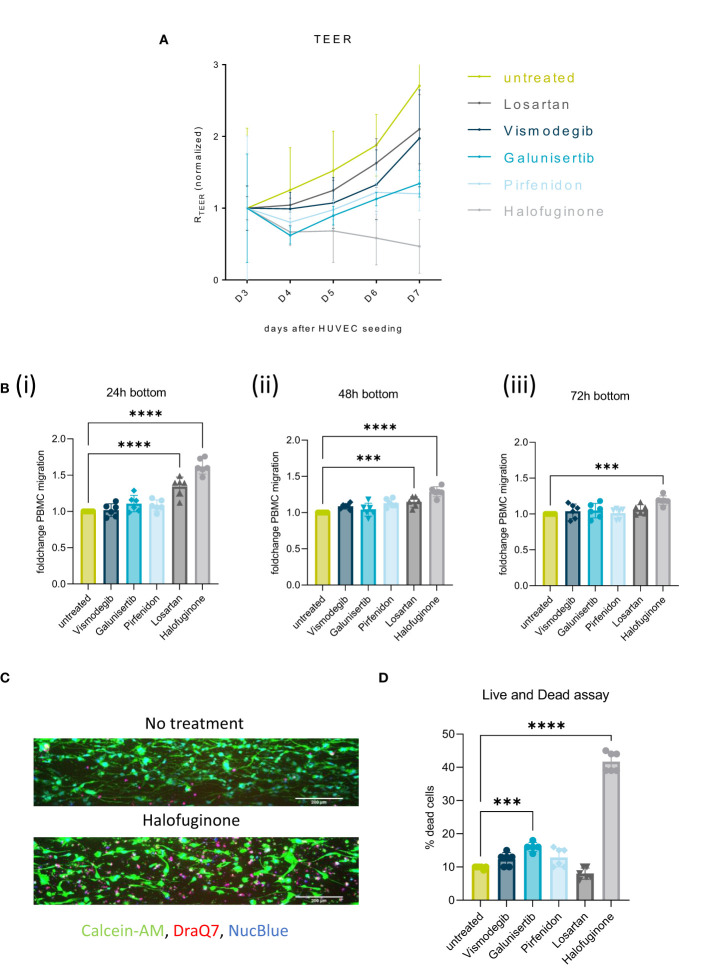
Stromal targeting in PDAC: **(A)** Barrier assessment of the culture setups when treated with 2 µM of either Vismodegib, Galunisertib, Pirfenidon, Losartan or Halofuginone with TEER. **(B)** Migration quantification of PBMCs migrated to the PDAC (bottom lane) at 24h(i), 48h(ii) and 72h(iii), when the stroma was treated with 2 µM of either Vismodegib, Galunisertib, Pirfenidon, Losartan or Halofuginone. The data, depicted as fold change, was normalized to the untreated control. Shown are mean +- SD (N=3, n=3). **(C)** Live and Dead assay with DraQ7 (red), Calcein-AM (green) and NucBLue (blue) showing the influence of the compounds on cell death when treated with Halofuginone in the PSC lane. The cells were stained with Calcein-AM, DraQ7 and NucBlue (N=3, n=3), imaged on the ImageXpress Micro Confocal (Molecular Devices). 10x acquisition, Scale bar=200 µm **(D)** Live and Dead assay quantification showing the percentage of dead cells compared to the total number of cells. ***p< 0,001; ****p< 0,0001.

To increase the amount of migrating PBMCs and consequent immune infiltrate into the tumor compartment, several stromal targeting compounds were tested. Halofuginone (PSC/CAF and SMAD2/3 inhibitor), Galunisertib (TGF-β receptor inhibitor), Pirfenidon (cell cycle inhibitor of CAFs), Losartan (TGF-β ligand inhibitor) and Vismodegib (SHH pathway inhibitor) were selected to target the PSCs compartment (**Figure 6**).

Exposure of the stromal compartment to these compounds decreased TEER, confirming that all compounds influenced PSCs barrier. Halofuginone created the biggest drop in TEER (**Figure 6A**). This effect was accompanied by a significant increase in immune cell infiltration in response to Halofuginone, Losartan and Pirfenidone (24-72h). Migration of PBMCs was slightly increased to the PDAC organoids in the bottom lane and the effect reduced over time (**Figure 6B**). Halofuginone produced the most consistent increase in immune cell infiltration. This seemed to be associated to the induction of pancreatic stellate cell death as determined with a live and dead assay (**Figures 6C, D**).

## Discussion

We here describe the development and the application of a PDAC microenvironment on-a-Chip model in immune cell migration and infiltration studies. Several model setups were established, these consisted of an endothelial tubule perfused with PBMCs, PSCs (stromal compartment), and PDAC organoids (**Figure 1**).

To study the influence of vascular permeability on PBMC migration, we simulated inflammation upon TNFα exposure to the HUVEC tubule. Endothelial tubules showed a clear morphological change (CD31) and increased ICAM expression in response to TNFα (**Figure 2A**). As a result, tubules showed a higher Papp (BI assay) and lower TEER measurements, suggesting that TNFα induced a weaker barrier function compared to untreated tubules (**Figures 2C, D**). TNFα improved PBMC attachment and subsequent transmigration (**Figure 2F**). Similar behavior was observed in presence of PSCs and PDAC organoids, and for this reason TNFα priming was not needed.

In the model setups composed by HUVEC-PSC-PDAC organoids, stromal cells formed a functional barrier on the interface with the endothelial tubule. 3D reconstruction images (**Figure 3A**) confirmed that PSCs align along the endothelial tubule and supported the formation of a physical barrier between the vascular and stromal compartments (**Figure 3B**). HUVECs tubules were leaky and seemed to give a minor contribution to the formation of this barrier, also demonstrated by similar TEER values measured at day 6 in triculture (HUVEC-PSCs-PDAC) and co-culture (PSCs-PDAC) (8) (**Figures 3B, C**). In presence of PSCs, 30% of migrating PBMCs were partly retained in the PSC compartment, suggesting that the stroma functioned like a barrier and influenced the distribution of part of the PBMC population and prevented its interaction with PDAC organoids (19).

However, two-three times as many cells reached the PDAC organoids compartment when PSCs were co-seeded with organoids in the bottom lane. PSCs seem to change when co-cultivated with PDAC organoids and formed a heterogenous stromal population. This stromal population likely included CAFs which in turn activate PDAC cells, promoting an increase in immunomodulatory chemokine secretion. PBMCs migration in our culture system seemed to depend on the formation of a chemokine gradient since no random migration was observed in absence of PSCs and/or PDAC over 72h (**Figure 3**). This was in line with previously published data by de Haan et al., 2021 where migration was only observed in presence of a chemokine gradient or a tumor compartment (14)​​. Total numbers of migrating cells indicated the importance of both PDAC organoids and PSCs in immune cell infiltration and distribution. Factors secreted in our on-chip model are normally associated to a negative effect on immune cell trafficking and infiltration into the tumor tissue (20–24)​. However, when the two cell types were put together, these seem to interact, secreting a higher concentration of several soluble factors (**Figure 4**). PDAC cells released CCL2, CXCL13 and IL-8, but in lower concentrations than secreted by the PSCs (**Figure 4**). High concentrations of CXCL1 were released, which is a chemoattractant for neutrophils and non-hematopoietic cells and is associated to immune escape programs (25). CCL4, a chemoattractant for NK-cells and monocytes associated to an immunosuppressive TME was increased (26)​​. CXCL13 plays a role in B cell recruitment, which we also confirmed with the migration data of B-cells, which show the highest migration potential (27) (**Figure 5**). Overall, PBMCs preferably migrated towards the PDAC organoids. However, direct immune cell-PDAC organoid interactions seemed to be partly prevented by the PSCs by the formation of a physical barrier as well as biochemical microenvironment that influenced immune cell distribution and did not support T cell migration (28). Due to the significant increase in chemokines, we hypothesize, that these serve as factors in immunosuppression, and thus explain the immune cell retention from the PDAC organoids (28).

To overcome the stromal barrier and to increase immune cell infiltration, stromal targeting compounds with different mechanisms of action were tested. All the tested compounds showed a decrease in TEER and barrier function. Halofuginone seemed to be the most promising candidate and Vismodegib the least (**Figure 6A**). Halofuginone increased immune cell infiltration after 24h and 48h towards PDAC organoids (bottom lane), indicating that this compound weakened stromal barrier function (**Figure 6B**). The effect was reduced after 48h, suggesting some sort of barrier regeneration and PSC proliferation.

Although this model provides a good insight into stromal functioning in PDAC, it poses limitations regarding complexity and full *in vivo* translatability. The employed system should evolve to incorporate matched donor materials. In addition, other components of the PDAC stroma should also be included such as CAFs, and its role characterized. Considering given limitations, this model could still be instrumental in the understanding of the formation of PDAC tumor immune infiltrate as well as how to potentially influence cellular therapies (e.g., CAR T cells, TCR engineered T Cells, TILs, NK cells) delivery and effectiveness (29)​.

## Conclusion

Recruitment of immune cells into the tumor tissue is an essential step that shapes the immune microenvironment and defines the ability of a tumor to respond or not to immune targeting strategies. In this study, a significant immunomodulatory role of the PDAC stromal compartment was characterized. This contributes to the formation of a physical barrier as well as the formation of PDAC biochemical microenvironment. As a result, interaction of immune and tumor cells was partly prevented. Stromal retention of immune cells was in part reversed by Halofuginone. In addition, the study showed the suitability of microfluidic platforms for generating complex models and recapitulating complex cellular interplay involved in the lack of an effective anti-tumor immune response in PDAC.

## Data availability statement

The original contributions presented in the study are included in the article/**Supplementary Material**. Further inquiries can be directed to the corresponding author.

## Ethics statement

Written informed consent from the donors for research use of the tissue was obtained prior to acquisition of the specimens. Tissues for the generation of models were collected under protocol number 55859, approved by the local Ethics Committee (Comitato Etico Azienda Ospedaliera Universitaria Integrata) to V.C. (Prog. 3456CESC, 27/09/21)”.

## Author contributions

Conceptualization: MG. Methodology: MG, L-MG. Investigation and analysis: MG., L-MG. Writing-draft preparation: MG. Writing- review and editing: MG, KQ, VC. Supervision: KQ. Organoid generation: VC, SA. All authors contributed to the article and approved the submitted version.

## Supplementary material

Supplementary Figure 1 Characterization of cells. **(A)**PDAC Pat. 39 organoids grown in Matrigel on a 6-well plate, 4x magnification. **(B)** PSC Klon 2.2 grown in Matrigel on a 6-well plate, 4x magnification. **(C)** Immunostaining of PDAC organoids with CK19 (red) and NucBlue (blue), 10x, imaged on the ImageXpress Micro Confocal (Molecular Devices). **(D)** Immunostaining of PSCs with Vimentin (red) and NucBlue (blue), 10x, imaged on the ImageXpress Micro Confocal (Molecular Devices).
