# Supplementary figures and images for: The tumor stroma influences immune cell distribution and recruitment in a PDAC-on-a-chip model
Source: Front Immunol. 2023 May 2;14:1155085. doi: 10.3389/fimmu.2023.1155085 (PMC10185841; doi:10.3389/fimmu.2023.1155085)

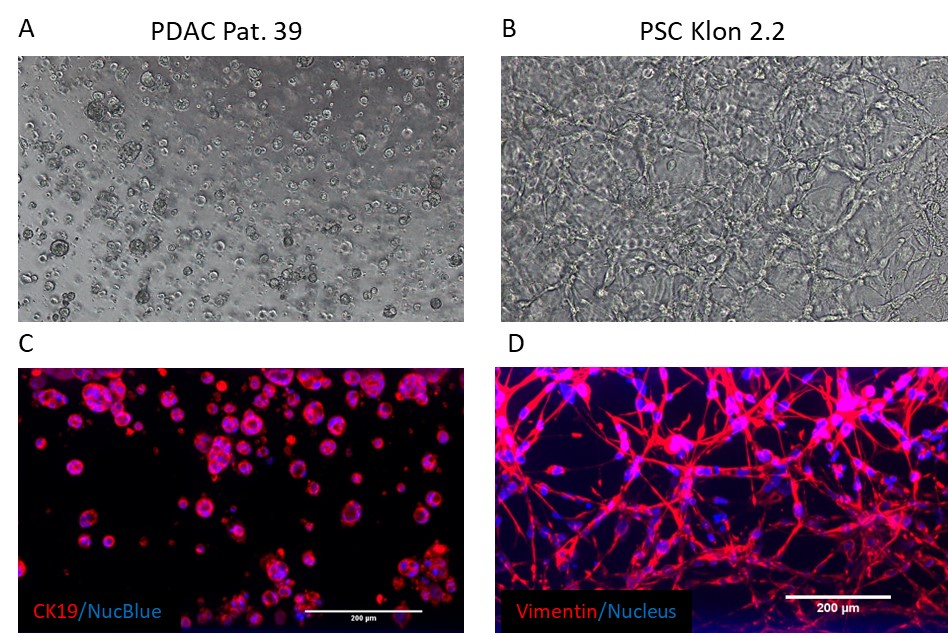

Supplement: Supplementary Figure 1 — Characterization of cells. (A)PDAC Pat. 39 organoids grown in Matrigel on a 6-well plate, 4x magnification. (B) PSC Klon 2.2 grown in Matrigel on a 6-well plate, 4x magnification. (C) Immunostaining of PDAC organoids with CK19 (red) and NucBlue (blue), 10x, imaged on the ImageXpress Micro Confocal (Molecular Devices). (D) Immunostaining of PSCs with Vimentin (red) and NucBlue (blue), 10x, imaged on the ImageXpress Micro Confocal (Molecular Devices). [file Image_1.jpeg]
